# Procurement of Deceased Donor Parathyroid Glands With the Aid of Near-infrared Autofluorescence Imaging

**DOI:** 10.1097/TXD.0000000000001306

**Published:** 2022-03-10

**Authors:** Casey J. Ward, Yvonne M. Kelly, Shareef M. Syed, Raphael P.H. Meier, Tadasuke Ando, Steven A. Wisel, James M. Gardner, Peter G. Stock, Quan-Yang Duh

**Affiliations:** 1 Division of Transplant Surgery, Department of Surgery, University of California San Francisco, San Francisco, CA.; 2 Department of Urology, Faculty of Medicine, Oita University, Yufu, Japan.

## Abstract

Parathyroid allotransplantation is a burgeoning treatment for severe hypoparathyroidism. Deceased donor parathyroid gland (PTG) procurement can be technically challenging due to lack of normal intraoperative landmarks and exposure constraints in the neck of organ donors. In this study, we assessed standard 4-gland exposure in situ and en bloc surgical techniques for PTG procurement and ex vivo near-infrared autofluorescence (NIRAF) imaging for identification of PTGs during organ recovery.

**Methods.:**

Research tissue consent was obtained from organ donors or donor families for PTG procurement. All donors were normocalcemic, brain-dead, solid organ donors between 18 and 65 y of age. PTGs were procured initially using a standard 4-gland exposure technique in situ and subsequently using a novel en bloc resection technique after systemic organ preservation flushing. Parathyroid tissue was stored at 4 °C in the University of Wisconsin solution up to 48 h post-procurement. Fluoptics Fluobeam NIRAF camera and Image J software were utilized for quantification of NIRAF signal.

**Results.:**

Thirty-one brain-dead deceased donor PTG procurements were performed by abdominal transplant surgeons. In the initial 8 deceased donors, a mean of 1.75 glands (±1.48 glands SD) per donor were recovered using the 4-gland in situ technique. Implementation of combined en bloc resection with ex vivo NIRAF imaging in 23 consecutive donors yielded a mean of 3.60 glands (±0.4 SD) recovered per donor (*P* < 0.0001). Quantification of NIRAF integrated density signal demonstrated >1-fold log difference in PTG (2.13 × 10^5^ pixels) versus surrounding anterior neck structures (1.9 × 10^4^ pixels; *P* < 0.0001). PTGs maintain distinct NIRAF signal from the time of recovery (1.88 × 10^5^ pixels) up to 48 h post-procurement (1.55 × 10^5^ pixels) in organ preservation cold storage (*P* = 0.34).

**Conclusions.:**

The use of an en bloc surgical technique with ex vivo NIRAF imaging significantly enhances the identification and recovery of PTG from deceased donors.

## INTRODUCTION

Hypoparathyroidism (hypoPT), defined by hypocalcemia from inappropriately low parathyroid hormone (PTH) levels, is currently one of the most common and debilitating complications of neck surgery worldwide. Transient postsurgical hypoPT lasting <6 mo is estimated to occur in 25.4% to 83% of patients worldwide after neck surgery,^1,2^ whereas permanent postsurgical hypoPT, defined as hypoPT lasting >6 mo, has been estimated to occur in approximately 0.12% to 4.6% of cases.^2,3^ In the United States alone, prevalence estimates project around 77 000 patients experience hypoPT.^4^ Emerging data have begun to elucidate the severe long-term sequelae of permanent hypoPT. A recent Scandinavian registry study found that permanent hypoPT patients had an increased risk for renal insufficiency (hazard ratio [HR], 4.88), for any malignancy (HR, 2.15), for cardiovascular events (HR, 1.88), and for death (HR, 2.09; 95% confidence interval, 1.04-4.20).^5^

In many patients, hypoPT is managed with regular use of calcium supplements and active vitamin D analogues, but a significant proportion of patients continue to experience severe symptoms requiring repeated emergency admissions and ultimately fail supplementation therapy alone.^6^ Furthermore, a recombinant PTH analogue has been shown to have limited clinical success, and the drug can cost up to $60 000 to $ 100 000 annually.^7^

A promising approach to treating permanent hypoPT is parathyroid allotransplantation. A wide range of case reports and methodologies have been described dating back to the 1950s with relative success.^8,9^ The most common source of viable parathyroid tissue for allotransplantation has come from hyperplastic glands of living donor patients with secondary hyperparathyroidism triggered by chronic renal failure.^9^ These glands were most often cryopreserved or cultured before transplantation, leading to a large degree of variance in allotransplantation strategies and outcomes.^8,9^ With respect to deceased donor sources of parathyroid tissue for allotransplantation, there are 3 case reports in the literature to date. In 1983, Sollinger et al described the outcomes of 2 patients transplanted with deceased donor parathyroid tissue from a cadaveric donor into the brachioradialis muscle with allograft function out to 16 mo.^10^ Chapelle et al described a combined deceased donor kidney/parathyroid transplantation for a patient with terminal renal failure and a previous total parathyroidectomy with excellent parathyroid allograft function.^11^ Most recently, Aysan et al reported a deceased donor parathyroid allotransplant with methylprednisolone induction and no maintenance immunosuppression with continued allograft function out to 36 mo.^12^

Because of the paucity of literature surrounding deceased donor parathyroid gland (PTG) procurement, this study’s aim was to describe our group’s initial learning curve with standard 4-gland exploration in situ and evolution toward en bloc cadaveric dissection ex vivo. Importantly, because of timing constraints related to concurrent recipient operations (notably heart, liver, and lung teams), the donor PTG procurement was performed after organ flushing, and all transplantable organs were procured. The evolution toward en bloc cadaveric dissection specifically addressed 3 major concerns related to cadaveric parathyroid procurement: (1) operative exposure constraints in the neck of organ donors due to postmortem concerns around funeral viewings; (2) significant change in tissue color and anatomical relationships between the PTG, thyroid gland, and lymph nodes in the cold phase of the donor operation; (3) any potential donation after circulatory death donor PTG procurements would be performed in the cold phase. These concerns are compounded by the fact that the recovering team consists of non-endocrine trained surgeons but rather abdominal transplant trained surgeons and organ/tissue procurement coordinators.

Additionally, we describe a new indication for PTG near-infrared autofluorescence (NIRAF) imaging in identifying normal parathyroid tissue in cadaveric donors. NIRAF imaging was adopted ex vivo because its use has decreased inadvertent resection of PTGs during thyroidectomy and lowered risk of postoperative hypocalcemia in randomized trials.^13,14^ Furthermore, we highlight the novel finding of parathyroid NIRAF signal stability for up to 48 h in cold storage.

## MATERIALS AND METHODS

### Research Consent and Organ Donor Selection

Research tissue consent was obtained from organ donors and donor families for PTG procurement. Donor selection included normocalcemic, brain-dead, solid organ donors between 18 and 65 y of age. The PTGs’ vascular supply was not included in cold perfusion circuit, and the glands were recovered after procurement of all intra-abdominal and intrathoracic organs to be used for clinical transplantation.

### Description of Deceased Donor Parathyroid Operation

#### Four-Gland In Situ PTG Procurement

Standard deceased donor procedures would occur, including warm dissection of thoracic and abdominal organs, administration of heparin, cannulation, aortic cross clamping, venting, ice cooling and cold dissection, and multiorgan sequential extraction. Vessel recovery would then be conducted. Once all standard organs and vessels had been extracted, the abdominal transplant procurement team would recover the PTGs. Our initial operative protocol involved replicating a standard 4-gland parathyroid exploration in situ in the first 8 donors. A standard collar incision was not utilized to avoid a visible incision during funeral viewing. Instead, due to these constraints, the standard donor sternotomy incision was only extended cephalad to the level of the cricoid cartilage. The overlying platysma and strap muscles were divided exposing the anterior portion of the thyroid. The superior pole of the thyroid was then fully mobilized bilaterally. The lateral aspects of the thyroid gland were then mobilized to allow medial and lateral rotation of the thyroid to expose the PTGs. Identified PTGs were then placed in cold University of Wisconsin (UW) solution for secondary confirmatory identification via histological examination. Additionally, the Fluobeam system was used ex vivo to confirm identification of candidate PTGs.

### NIRAF Imaging

Fluoptics Fluobeam camera and software were utilized for all PTG NIRAF imaging using high-sensitivity factory settings. NIRAF imaging was performed within 6 h of procurement and after 48 h of cold storage in the UW solution. PTGs identified by Fluobeam were confirmed by frozen section histology. After frozen sections were cut, hematoxylin and eosin staining was performed per the University of California San Francisco Department of Pathology protocols.

### Quantitative Analysis of NIRAF Images and Statistical Analysis

NIRAF images of en bloc specimens obtained on the Fluobeam platform were uploaded directly to the Image J software (US National Institutes of Health) for quantification of the integrated density (the sum of the value of pixels in a defined selection area). Equivalently sized regions of interest in the thyroid gland and anterior neck structures (5-mm diameter) were compared. Example images of quantification methodology are illustrated in Figure S1, SDC, http://links.lww.com/TXD/A407.

Two-tailed unpaired *t* test and Mann-Whitney *U* test were performed using the Prism GraphPad Pro (San Diego, CA) software.

## RESULTS

### Initial Experience Using 4-Gland In Situ PTG Procurement

**FIGURE 1.**
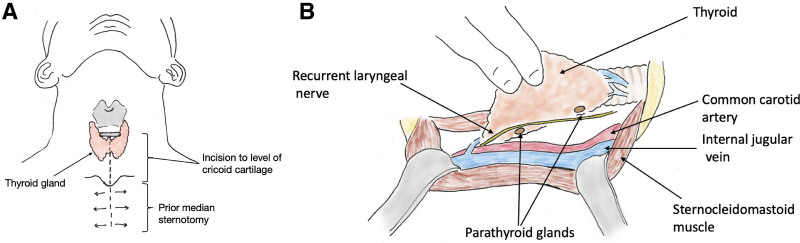
Deceased donor parathyroidectomy operation: initial experience. A, The standard donor sternotomy incision was extended cephalad to the level of the cricothyroid cartilage. B, The superior pole of the thyroid was taken down sharply to allow medial and lateral rotation of the thyroid to expose the parathyroid glands.

From January 2018 to February 2019, 31 brain-dead deceased donor PTG procurements were performed by abdominal transplant recovery teams. In our initial experience in 8 donors, we replicated a standard 4-gland exploratory technique in situ through the sternotomy incision during cold phase of donor procurement (Methods; Figure 1A and B). In the initial 8 donors, we identified a mean of 1.75 glands (±1.48 glands SD) per donor. Candidate PTGs were confirmed via NIRAF imaging and histological confirmation. The average time for parathyroid extraction was >60 min.

### Transition to En Bloc Resection With NIRAF Imaging

Persistent difficulty locating normal PTGs using the standard 4-gland exploratory technique in situ was due to suboptimal exposure, inexperience of recovering surgeons (non-endocrine surgeons), and a significant change in the distinctive color differences between the PTG, thyroid gland, and lymph node tissue in the cold phase of the operation. An evolution to en bloc resection of the thyroid, parathyroids, and surrounding anterior cervical structures was adopted in the following 23 consecutive donors. The anatomic borders of the en bloc resection can be found in Table 1. The recurrent laryngeal nerve was divided without attempting identification as part of the en bloc resection.

**TABLE 1.** Anatomic boundaries of anterior cervical en bloc resection

Right lateral border	Right common carotid/brachiocephalic artery
Left lateral border	Left common carotid artery
Inferior border	Aortic arch
Superior border	Superior thyroid artery and vein pedicle
Posterior border	Prevertebral (lateral); pretracheal layer (medial)
Anterior border	Infrahyoid muscles

**FIGURE 2.**
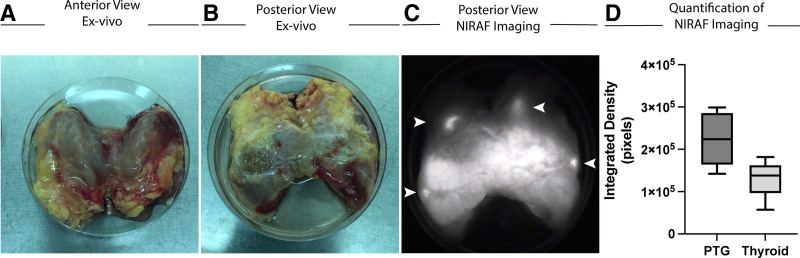
Procurement of parathyroid glands (PTGs) from deceased donors is greatly facilitated by near-infrared autofluorescence (NIRAF) imaging. Anterior (A) and posterior (B) views of en bloc procured anterior cervical tissue including thyroid and surrounding structures. C, Fluobeam-obtained NIRAF image (posterior view, same as Figure 2B) of en bloc specimen indicating 4 distinct NIRAF imaging signatures correlating with PTGs (marked by white arrows). D, Quantification of NIRAF integrated density signal showing a nearly 2-fold significant increase in PTG compared with thyroid.

En bloc tissue was procured after systemic organ preservation flushing and placed in cold UW preservation solution (Figure 2A and B). Average en bloc extraction time was 15 to 30 min when performed by abdominal transplant surgeons and ultimately by organ/tissue procurement coordinators.

NIRAF imaging was then implemented ex vivo using the Fluobeam platform to identify candidate PTGs (Figure 2C and D). After en bloc extraction and ex vivo NIRAF imaging implementation, in 23 consecutive donors, at least 3 glands were procured in all patients and 4 were procured in 14 of 23 donors. A mean of 3.60 glands (±0.4 SD) per donor (*P* < 0.0001) were identified and confirmed by histological confirmation.

### Quantitative Analysis of NIRAF Images

Using NIRAF images from 3 consecutive donor en bloc specimens, we quantified the integrated density of 6 PTGs and 9 equivalently sized regions of interest (5-mm diameter) in the thyroid gland and showed there is a significant increase in the integrated density signal in PTGs (2.24 × 10^5^ pixels; SEM, ±2.8 × 10^4^) compared with thyroid tissue (1.3 × 10^5^ pixels; SEM, ±1.3 × 10^4^; *P* < 0.01; Figure 2D).

**FIGURE 3.**
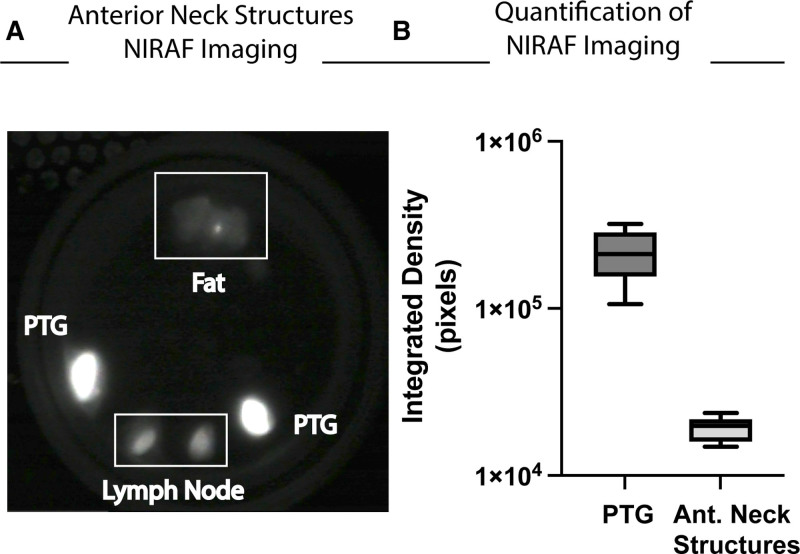
Near-infrared autofluorescence (NIRAF) imaging of deceased donor parathyroid glands (PTGs) is distinct ex vivo from surrounding anterior (Ant.) neck structures. A, Comparison of NIRAF signal intensity of Ant. neck structures including fat, cervical lymph node, and PTG from the same donor. B, Quantification of NIRAF integrated density signal showing a >1-fold log significant difference in PTG vs surrounding Ant. neck structures.

After dissection of candidate PTGs from en bloc specimens, we then quantified the integrated density of NIRAF images of PTGs versus equivalently sized (5 mm) surrounding anterior neck structures (fat, lymph nodes, thyroid tissue). Using 4 different donors, we compared 14 PTGs versus 9 anterior neck structures and showed there is a significantly >1-fold log difference in integrated density signal in PTG (2.13 × 10^5^ pixels; SEM, ±1.88 × 10^4^) versus other anterior neck structures (1.9 × 10^4^ pixels; SEM, ±1.04 × 10^3^; *P* < 0.0001; Figure 3B).

**FIGURE 4.**
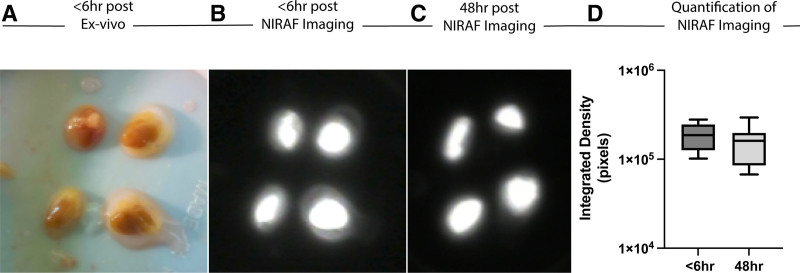
Near-infrared autofluorescence (NIRAF) imaging of deceased donor parathyroid glands (PTGs) is stable ex vivo. A, PTGs were procured and isolated with (B) images taken using the Fluobeam NIRAF platform <6 h post-procurement and (C) 48 h after cold storage in University of Wisconsin organ preservation solution. D, Quantification of NIRAF integrated density signal showing no significant difference in PTG at <6 vs 48 h time points post-procurement.

Furthermore, in 2 consecutive donors, the integrated density signal of 8 PTGs remained quantitatively stable from time of initial NIRAF imaging (1.88 × 10^5^ pixels; SEM, ±2.22 × 10^4^) compared with after 48 h in cold storage UW preservation solution (1.55 × 10^5^ pixels; SEM, ±2.92 × 10^4^; *P* = 0.34; Figure 4D).

## DISCUSSION

PTG NIRAF imaging is an emerging technology used intraoperatively to enhance identification and preservation of PTGs during thyroid, parathyroid, and anterior neck surgical procedures.^15-17^ In up to 30% to 67% of cases, NIRAF imaging has been shown to aid the operating surgeon in locating previously unrecognized PTGs intraoperatively due to their characteristic NIRAF signature.^15^ The unique NIRAF signature of PTG allows for its identification, especially when normal intraoperative visual cues are not available to the surgeon, as exemplified during deceased donor en bloc PTG procurement.^18^ As shown in Figure 2A and B, our groups transition to en bloc resection distorted the visual cues used to identify normal PTGs. Importantly, our evolution in surgical technique to en bloc resection also allowed for non-endocrine surgery trained individuals, abdominal transplant surgeons, and eventually organ/tissue coordinators, to reliably replicate the cadaveric en bloc PTG procurement technique in an efficient manner, without interrupting the standard donor procedure.

Our data further corroborate the ability of NIRAF imaging to help the surgeon differentiate PTGs from other anterior neck structures including fat, lymph nodes, and thyroid tissue that often make PTG identification difficult (Figures 2D and 3B). In addition, the PTG’s unique NIRAF signal (a near-infrared signature still unknown to date) is also qualitatively stable for up to 48 h of storage at 4 °C in UW organ preservation solution (Figure 4D). Of note, although we quantified the integrated density signal of Fluobeam-obtained NIRAF, this is not a clinically approved standard operating procedure and is not the current standard practice in the field of endocrine surgery.

We chose to utilize NIRAF imaging as it provided a large overall view and is best used by the surgeon in searching for candidate PTGs without needing to specify the exact location of the tissue. In contrast, another FDA-approved device to identify PTGs intraoperatively using NIRAF is PTeye. Instead of imaging, the PTeye device uses a probe that measures NIRAF signal upon contact with parathyroid candidate tissue. Parathyroid tissue emits approximately 7 to 10× higher NIRAF than the thyroid background (when threshold is set at 1.2). False positive signals can still occur in some tissues such as brown fat, colloid nodules, and thyroid cancer. Although this study utilized only a NIRAF imaging platform (eg, Fluobeam), we believe a NIRAF probe platform (eg, PTeye) could be used interchangeably or synergistically to identify parathyroid candidates ex vivo. NIRAF probe has the advantage of quantitative measurement, meaning it could be used to pinpoint (instead of searching for) parathyroid tissue.^19^ Ultimately, once the PTG is identified, it should be confirmed by measuring the PTH level in needle aspirate or culture medium^20^ or histologically before transplantation.

Limitations of this study include that due to the institutional review board and organ procurement organization (OPO) constraints, a standard collar incision utilized for most standard thyroid operations was not allowed due to potential cosmetic reasons postmortem. Additionally, PTGs were recovered in the cold phase after all transplantable organs were retrieved in conjunction with local OPO policies. The potential recovery of PTG during the warm phase of the donor operation with the presence of formally trained endocrine surgeons using a standard collar incision in cooperation with local OPO policies may lead to more consistent yield of deceased donor PTGs. This would require future study and policy changes for full practical implementation with current donor operation logistics.

Along with our group’s recently described novel application of whole PTG cotransplantation to improve pancreatic islet engraftment and diabetes reversal (Pancreatic Islets and Parathyroid Gland Co-Transplantation for Treatment of Type 1 Diabetes; https://www.clinicaltrials.gov; unique identifier: NCT03977662; registered in 2019), we believe the renewed and emerging interest in exploring parathyroid allotransplantation as a treatment for permanent hypoPT will require a thorough understanding of PTG procurement techniques and storage and viability practices for potential allotransplantation protocols.^21^

In summary, this study describes our group’s learning curve and surgical technique for enhanced identification of normal cadaveric PTGs via en bloc resection and ex vivo NIRAF imaging. We believe the standardization of a deceased donor PTG procurement technique with the aid of NIRAF imaging will have vast implications for future basic research of normal PTGs and potential allogeneic transplantation trials of PTG for hypoPT and cotransplantation with pancreatic islets for type 1 diabetes.

## Supplementary Material


