# Supplementary material for: Procurement of Deceased Donor Parathyroid Glands With the Aid of Near-infrared Autofluorescence Imaging
Source: Transplant Direct. 2022 Mar 10;8(4):e1306. doi: 10.1097/TXD.0000000000001306 (PMC8923582; doi:10.1097/TXD.0000000000001306)
Supplement: Supplementary file 1 [file txd-8-e1306-s001.pdf]

Supplemental Digital Content 7. Categorical outcomes, Unplanned repeat surgeries for revision, Unplanned repeat surgeries for complications, Pain, Infections, and Reconstructive failure

Study, Year, PMID, Country	Overall RoB	Outcome	Outcome Description	Time Point	Group	Subgroup	n/N (%)	Adjusted Odds Ratio (95% CI)	P Value
Fischer, 2014, 24916480, US	High	Unplanned repeat surgery for revision	Unplanned revision	<6 mo	IBR	All	8/155 (5.2)	Ref	Ref
	High	Unplanned repeat surgery for revision	Unplanned revision	<6 mo	AR	All	6/155 (3.9)	NR	0.56
	High	Unplanned repeat surgery for revision	Unplanned revision	<1 y	IBR	All	17/155 (11.0)	Ref	Ref
	High	Unplanned repeat surgery for revision	Unplanned revision	<1 y	AR	All	7/155 (4.5)	NR	0.017
	High	Unplanned repeat surgery for revision	Unplanned revision	<2 y	IBR	All	21/155 (13.5)	Ref	Ref
	High	Unplanned repeat surgery for revision	Unplanned revision	<2 y	AR	All	7/155 (4.5)	NR	0.003
Kulkarni, 2017, 28713853, US & Canada	Moderate	Unplanned repeat surgery for revision	Unplanned revision	2 y	IBR DTI	All	31/93 (33.3)	vs. IBR with TE: 0.58 (0.35, 0.96)	0.035
	Moderate	Unplanned repeat surgery for revision	Unplanned revision	2 y	IBR TE	All	503/1263 (39.8)	Ref	Ref
	Moderate	Unplanned repeat surgery for revision	Unplanned revision	2 y	AR with DIEP	All	223/350 (63.7)	vs. IBR with TE: 2.66 (1.83, 3.86)	<0.001
	Moderate	Unplanned repeat surgery for revision	Unplanned revision	2 y	AR with free TRAM	All	56/87 (64.4)	vs. IBR with TE: 2.26 (1.35, 3.78)	0.002
	Moderate	Unplanned repeat surgery for revision	Unplanned revision	2 y	AR with pedicled TRAM	All	40/77 (57.1)	vs. IBR with TE: 1.34 (0.75, 2.40)	0.33
	Moderate	Unplanned repeat surgery for revision	Unplanned revision	2 y	AR with LD	All	41/64 (64.1)	vs. IBR with TE: 1.97 (1.07, 3.64)	0.031
	Moderate	Unplanned repeat surgery for revision	Unplanned revision	2 y	AR with SIEA	All	33/62 (53.2)	vs. IBR with TE: 1.83 (0.93, 3.60)	0.079
Zhang, 2019, 30675702, China	High	Unplanned repeat surgery for revision	Unplanned reoperation	4.9 y	IBR	All	230/394 (58.4)	Ref	Ref
	High	Unplanned repeat surgery for revision	Unplanned reoperation	4.9 y	AR	All	154/438 (35.2)	vs. IBR: 0.72 (0.50, 1.06)	0.093

Study, Year, PMID, Country	Overall RoB	Outcome	Outcome Description	Time Point	Group	Subgroup	n/N (%)	Adjusted Odds Ratio (95% CI)	P Value
Hangge, 2013, 31606126, US	High	Unplanned repeat surgery for complications	NR	NR	IBR DTI	All	81/193 (42)	vs. AR: 2.03 (1.03, 3.98)	0.042
	High	Unplanned repeat surgery for complications	NR	NR	IBR TE	All	58/146 (40)	vs. AR: 1.81 (0.90, 3.64)	0.096
	High	Unplanned repeat surgery for complications	NR	NR	AR	All	17/60 (28)	Ref	Ref
Mioton, 2013, 23562485, US	Moderate	Unplanned repeat surgery for complications	NR	1 mo	IBR	All	662/9786 (6.76)	1.08 (0.88, 1.32)	NR
	.	Unplanned repeat surgery for complications	NR	1 mo	AR	All	316/3296 (9.59)	Ref	Ref
Zhang, 2019, 30675702, China	High	Unplanned repeat surgery for complications	Urgent surgery for a compromised implant/flap	4.9 y	IBR	All	31/394 (7.9)	vs. AR: 0.63 (0.29, 1.37)	NR
	High	Unplanned repeat surgery for complications	Urgent surgery for a compromised implant/flap	4.9 y	AR	All	33/438 (7.5)	Ref	Ref
de Araujo, 2016, 27673527, US	High	Infections	SSI	4.3 y	IBR	All	NR/38 (NR)	vs. AR 0.86 (0.18, 4.11)	0.847
	High	Infections	SSI	4.3 y	AR	All	NR/32 (NR)	Ref	Ref
Garvey, 2012, 23096600, US	Moderate	Infections	Infections	NR	IBR	All	50/442 breasts (11.3)	Ref	Ref
	Moderate	Infections	Infections	NR	AR	All	21/548 breasts (3.8)	vs. IBR: NR	<0.001
Kulkarni, 2017, 28713853, US & Canada	Moderate	Infections	Breast WI	2 y	IBR	All	NR	NR	NR
	Moderate	Infections	Breast WI	2 y	IBR DTI	All	17/112 (15.2)	vs IBR TE: 1.70 (0.91, 3.18)	0.1
	Moderate	Infections	Breast WI	2 y	IBR TE	All	159/1525 (10.4)	Ref	Ref
	Moderate	Infections	Breast WI	2 y	AR (all)	All	NR	NR	NR
	Moderate	Infections	Breast WI	2 y	AR with DIEP	All	27/390 (6.9)	vs IBR TE: 0.44 (0.25, 0.78)	0.005
	Moderate	Infections	Breast WI	2 y	AR with free TRAM	All	5/95 (5.3)	vs IBR TE: 0.45 (0.17, 1.18)	0.10
	Moderate	Infections	Breast WI	2 y	AR with pedicled TRAM	All	8/85 (9.4)	vs IBR TE: 0.73 (0.31, 1.70)	0.46
	Moderate	Infections	Breast WI	2 y	AR with LD	All	6/71 (8.5)	vs IBR TE: 0.50 (0.15, 1.56)	0.23

Study, Year, PMID, Country	Overall RoB	Outcome	Outcome Description	Time Point	Group	Subgroup	n/N (%)	Adjusted Odds Ratio (95% CI)	P Value
	Moderate	Infections	Breast WI	2 y	AR with SIEA	All	8/65 (12.3)	vs IBR TE: 0.67 (0.25, 1.82)	0.43
Mioton, 2013, 23562485, US	Moderate	Infections	WI	1 mo	IBR	All	338/9786 (3.45)	Ref	Ref
	Moderate	Infections	WI	1 mo	AR	All	180/3296 (5.46)	vs IBR: 1.40 (1.01, 1.96)	NR
	Moderate	Infections	Superficial SSI	1 mo	IBR	All	163/9786 (1.67)	Ref	Ref
	Moderate	Infections	Superficial SSI	1 mo	AR	All	97/3296 (2.95)	vs IBR: 1.20 (0.81, 1.76)	NR
	Moderate	Infections	Deep SSI	1 mo	IBR	All	195/9786 (1.07)	Ref	Ref
	Moderate	Infections	Deep SSI	1 mo	AR	All	65/3296 (1.97)	vs IBR: 1.81 (1.12, 2.94)	NR
Naoum, 2020a, 31756414, US	High	Infections	NR	4-10 y	IBR	All	23/633 breasts (3.6)	Ref	Ref
	High	Infections	NR	4-10 y	AR	All	9/342 breasts (2.6)	vs IBR: 0.77 (0.20, 2.50)	0.67
Naoum, 2020b, 32607638, US	High	Infections	NR	4.3-6.3 y	IBR DTI	All	7/127 (5.5)	Ref	Ref
	High	Infections	NR	4.3-6.3 y	IBR with TE	All	2/88 (2.2)	Ref	Ref
	High	Infections	NR	4.3-6.3 y	AR	All	11/85 (13.0)	vs. IBR DTI: 3.2 (0.6, 16)<br>vs. IBR with TE: 8.1 (1.7, 39)	0.20<br>0.009
Chetta, 2017, 28002254, US	High	Reconstructive failure	NR	1.3 mo	IBR	All	1101/3746 (29.4)	Ref	Ref
	High	Reconstructive failure	NR	1.3 mo	AR	All	40/935 (4.3)	vs IBR: 0.09 (0.07, 0.13)	<0.001
Fischer, 2013, 23629074, US	High	Reconstructive failure	Flap loss in AR or unplanned, nonaesthetic TE/I removal related to a complication in IBR	NR	IBR	All	4/60 (7.3)	Ref	Ref
	High	Reconstructive failure	Flap loss in AR or unplanned, nonaesthetic TE/I removal related to a complication in IBR	NR	AR	All	2/142 (1.3)	vs IBR: 0.19 (0.04, 0.80)	0.03

Study, Year, PMID, Country	Overall RoB	Outcome	Outcome Description	Time Point	Group	Subgroup	n/N (%)	Adjusted Odds Ratio (95% CI)	P Value
Garvey, 2012, 23096600, US	Moderate	Reconstructive failure	NR	NR	IBR	All	70/442 breasts (15.8)	Ref	Ref
	Moderate	Reconstructive failure	NR	NR	AR	All	8/548 breasts (1.5)	vs IBR: NR	<0.001
Kulkarni, 2017, 28713853, US & Canada	Moderate	Reconstructive failure	NR	2 y	IBR (all)	All	NR	Ref	Ref
	Moderate	Reconstructive failure	NR	2 y	IBR (all)	Unilateral	41/600 (6.83)	NR	NR
	Moderate	Reconstructive failure	NR	2 y	IBR (all)	Bilateral	74/994 (7.44)	NR	NR
	Moderate	Reconstructive failure	NR	2 y	AR (all)	All	NR	NR	NR
	Moderate	Reconstructive failure	NR	2 y	AR (all)	Unilateral	4/317 (1.26)	vs. IBR (all): 0.12 (0.04, 0.36)	<0.001
	Moderate	Reconstructive failure	NR	2 y	AR (all)	Bilateral	4/224 (1.87)	vs. IBR (all): 0.14 (0.05, 0.45)	0.001
Mioton, 2013, 23562485, US	Moderate	Reconstructive failure	Implant or flap failure	1 mo	IBR	All	83/9786 (0.85)	Ref	Ref
	Moderate	Reconstructive failure	Implant or flap failure	1 mo	AR	All	10/3296 (3.13)	vs. IBR: 1.69 (1.08, 2.62)	NR

Abbreviations: AR = autologous reconstruction, CI = confidence interval, DVT = deep vein thrombosis, ED = emergency department, IBR = implant-based reconstruction, N/A = not applicable, NR = not reported, NRCS = nonrandomized comparative study, PE = pulmonary embolism, PMID = PubMed identifier, RoB = risk of bias, SSI = surgical site infection, y = years.
